# Mechanism of Beraprost Effects on Pulmonary Hypertension: Contribution of Cross-Binding to PGE2 Receptor 4 and Modulation of O_2_ Sensitive Voltage-Gated K^+^ Channels

**DOI:** 10.3389/fphar.2018.01518

**Published:** 2019-01-18

**Authors:** Fenling Fan, Hua Tian, Jie Geng, Jizhao Deng, Ya Liu, Chunyan Chen, Songlin Zhang, Yushun Zhang, Jie Li, Hongyan Tian, Anthony M. Dart, Yuliang Zou

**Affiliations:** ^1^Department of Cardiovascular Medicine, The First Affiliated Hospital of Xi’an Jiaotong University, Xi’an, China; ^2^Baker Heart and Diabetes Institute, Melbourne, VIC, Australia; ^3^Department of Respiratory, The First Affiliated Hospital of Xi’an Jiaotong University, Xi’an, China; ^4^Department of Gynecology and Obstetrics, The First Affiliated Hospital of Xi’an Jiaotong University, Xi’an, China; ^5^Department of Cardiovascular Medicine, The Alfred Hospital, Melbourne, VIC, Australia

**Keywords:** pulmonary arterial hypertension, prostanoids, Beraprost, IP receptor, EP4 receptor, Kv channel, O_2_- sensitive Kv channel

## Abstract

**Background:** The purpose of this study is to elucidate mechanism(s) by which the orally active PGI2 analog, Beraprost (BPS), ameliorates pulmonary hypertension (PH). Prostaglandins are an important treatment for PH. Mechanisms of their action are not fully elucidated in relation to receptor subtype and effects on O_2_ sensitive Kv channels.

**Methods:** Distal (3rd order and beyond) pulmonary arteries from chronically hypoxic rats and from humans with established PH were studied. Measurements included pulmonary haemodynamics and histology, vascular reactivity, prostanoid receptor expression and activity of the O_2_ sensitive Kv channels.

**Results:** Prostacyclin receptor (IP), prostaglandin receptor E3 (EP3) and prostaglandin receptor E4 (EP4) are the main pulmonary artery receptor subtypes in both rat and human pulmonary arteries. Circulating levels of PGI2 and PGE2 were reduced in PH. PH was also associated with reduced receptor expression of IP but not of EP4. The effects on IP expression were overcome with BPS. Dilatory responses in PH to BPS were reduced in the presence of EP4 blockade. Expression and activity of oxygen sensitive Kv channels were reduced in pulmonary artery smooth muscle cell from rats with PH and humans with PAH and were also overcome by administration of BPS. Effects of BPS on oxygen sensitive Kv channels were reduced in the presence of EP4 blockade implicating the EP4 receptor, as well as the IP receptor, in mediating BPS effects.

**Conclusion:** Reduced expression of pulmonary IP receptors and reduced activity of O_2_ sensitive Kv channels are found in PH in both humans and rats. The orally active prostacyclin analogue, BPS, is able to reverse these changes, partly through binding to the EP4 receptor.

## Background

Pulmonary hypertension is a chronic, progressive disease characterized by elevation of PAP, ultimately resulting in right ventricular failure and death. One cause of PH, PAH may be idiopathic or familial but can also arise from underlying disorders such as connective tissue diseases and congenital heart disease (CHD) (Archer et al., 2010). Prognosis has substantially improved recently from treatments targeting three signaling pathways (prostacyclin, endothelin-1 or nitric oxide), and leading to current 1-year survival rates of 85–97% (Thenappan et al., 2007; Lau et al., 2017). Prostacyclin based therapies are a mainstay of contemporary treatment and have been shown in a short term trial to improve survival (Barst et al., 1996). Although a number of GPCR are prostanoid receptors (Breyer et al., 2001), the main biological target for PGI2 and analogues is thought to be the IP receptor with Gs coupling leading to activation of AC and formation of cyclic AMP resulting in vasodilation (Mubarak, 2010). BPS, as with other prostacyclin analogs, acts primarily on vascular rather than circulating cells (Humbert and Ghofrani, 2016). At lower affinity there is also binding to the EP4 receptor also resulting in AC activity (Sugimoto and Narumiya, 2007). PGI2 also binds to the EP1 receptor resulting in activation of Gq/Gi with subsequent reduction in cyclic AMP or elevation in Ca^2++^ (Gomberg-Maitland and Olschewski, 2008). Interestingly differences between prostacyclin analogues in terms of binding affinities have been observed (Whittle et al., 2012). Previous studies have shown that Iloprost binds to IP, EP1, EP3, and EP4 whereas treprostinil binds to EP2 as well as EP3 (Whittle et al., 2012; Humbert and Ghofrani, 2016). BPS is the only orally administered prostacyclin analogue that has been approved for the treatment of PAH (in Japan and South Korea). However, little is known about the binding affinities of BPS with prostaglandin receptors in PAH, in particular in relation to cross binding with non-IP receptors.

Some voltage-gated K^+^ channels (Kv) in peripheral pulmonary arteries are known to be O_2_-sensitive (Hulme et al., 1999). Thus the heterologously expressed Kv1.2, 1.5, 2.1, and 3.1 open in response to an increase in O_2_ tension. Expressions of Kv1.5, 2.1, and 1.2 are decreased in hypoxia induced PH in rats and expression of Kv1.5 is also decreased in human PASMCs of PAH patients (Wang et al., 1997). Thus it has been hypothesized that PAH may result from deficiency in the O_2_-sensitive Kv channels of PASMC (Hulme et al., 1999). Such O_2_ sensitive channels are known to be particularly expressed in resistance vessels (Archer et al., 2004). This also raises the possibility that the effects of PGI2 and analogues may be mediated, at least in part, by effect on Kv channels. A more complete understanding of the effect of prostaglandin, including on Kv channel, in PAH is important for the development of improved therapies. Therefore, in this study, the relationship between prostaglandin pathways and O_2_-and 4-AP-sensitive, voltage-gated K^+^ channels (Kv) including Kv1.5, Kv1.2, and Kv2.1, and the effect of BPS on these Kv channels were determined.

We present data from humans with PH as well as animals exposed to chronic hypoxia. Whilst not reproducing all the features of human PH, chronic hypoxia is one of the most widely used experimental paradigms in this field (Long et al., 2009; Stenmark et al., 2009; Cahill et al., 2012).

## Materials and Methods

### Study Groups and Tissue Collection

The use of the animals was approved by the Animal Experimentation Committee of Medical College, Xi’an Jiaotong University. Adult male Sprague-Dawley (SD) rats (350–400 g body wt) were housed under controlled temperature (22°C) and lighting (12/12-h light/dark cycle) conditions, with free access to food and water. Animals were randomly divided into three groups; Control (*n* = 12), Experimental PH (*n* = 24) and Experimental PH treated with BPS (*n* = 12). Experimental PH rats were placed in an automated normal pressure low oxygen chamber with oxygen concentration of 10 ± 1% for 8 h a day for 4 weeks as previously described (Xue, 1989).

Age and gender matched control rats were placed in normal air chamber (Control). A subgroup of the hypoxia rats were medicated with BPS (Experimental PH+BPS). BPS was administered intragastrically at a dose of 300 μg/kg a day for 27 days starting on the second day of hypoxic exposure. Control rats and experimental PH rats not receiving BPS were intragastrically administered 5 μg/kg/day of 0.9% saline.

Pulmonary artery pressure, right ventricular weight (RV), left ventricular (LV) weight and ventricular septum weight (S) were measured to estimate the RVHI. After animals had been sacrificed, RV was separated from the LV and S, and each of them was weighed. RV hypertrophy index was calculated as follows: [RV/(LV + S)] (Liu et al., 2013). Pulmonary artery (PA) tissues from the third and subsequent bifurcations of the pulmonary arterial tree of each experimental animal were immediately frozen in liquid nitrogen and then stored at -70°C for further experiments, or immediately placed in relevant reagents according to experimental demands. Single separate tissue samples were used for each experiment as described.

#### Human Tissue Specimens

Specimens were obtained from 11 PAH patients (five male, six female, age 49 ± 10 years old) undergoing open chest CHD repair and 12 patients (six male, six female, age 52 ± 10 years old) with lung cancer. Patient characteristics are shown in Table 1. All patients were receiving PAH therapy prior to repair: four were receiving monotherapy (two were receiving Sildenafil, and one each Bosentan or Beraprost), four were receiving initial combination therapy (Sildenafil and Bosentan or Sildenafil, Bosentan and Treprostinil). The remaining three patients had received sequential therapy from monotherapy to combination therapy (one from Sildenafil to Sildenafil and Bosentan and two from Beraprost to Beraprost and Bosentan). A small piece of lung tissue from the right lobe was collected from each patient. PA branches from those lung tissue segments were dissected free as soon as possible, frozen in liquid nitrogen and then stored at -70°C for further experiments.

**Table 1:** Patient characteristics.

Parameters	PAH	Non-PAH
N	11	12
Age	49 ± 10	52 ± 10
Female (*n*)	6	6
WHO Functional Class II/III/IV (*n*)	3/5/4	5/4/2
6 Min Walk Distance (m)	302 ± 80^∗∗^	520 ± 120
Hb, g/L	128 ± 64	104 ± 42
PaCO2 (A, mm Hg)	28 ± 11.3	36 ± 12.8
PaO2 (A, mm Hg)	81.9 ± 11.3	82.4 ± 12.4
SaO2 (A), (%)	94.2 ± 14.7	96.8 ± 15.2
Right Atrial Pressure, mmHg	6.7 ± 4.0^∗∗^	1.7 ± 0.5
Mean Pulmonary Artery Pressure, mmHg	47 ± 12.5^∗∗^	16 ± 8.2
Pulmonary Vascular Resistance, dyn-s/cm5	368.7 ± 102.4^∗∗^	146.4 ± 62.2
Pulmonary Artery Wedge Pressure, mmHg	10.6 ± 3.2	11.4 ± 3.0
Cardiac Output, L/min	3.2 ± 1.6^∗^	4.6 ± 1.8
FEV1, L/s	2.0 ± 0.6	1.9 ± 0.6
FVC, % predicted	76.3 ± 19.1	82.6 ± 19.8
FEV1/FVC	0.8 ± 0.1	0.8 ± 0.1
Total lung capacity, L	4.1 ± 1.2	4.2 ± 1.1

*The table shows patient demographics, functional measures, pulmonary artery blood gas and haemodynamic measures (from right heart catheterization) and lung function. Results are mean ± SD or n. ^∗^*p* < 0.05, ^∗∗^*p* < 0.01 PAH vs. Non-PAH.*

Written informed consent was obtained from all patients. The study was approved by the ethics committee of the First Affiliated Hospital of Xi’an Jiaotong University.

### Tension Measurements

Measurements of vessel tension of isolated distal PA rings from the third and sub-sequent branches was performed in all animals (Thebaud et al., 2002). The isolated PAs from control rats were immediately placed in an organ bath with oxygenated Krebs solution, while PA rings from Experimental PH rats were placed in hypoxic Krebs solution (PO_2_ = 10 ± 1 mmHg). Dilatation dose-response curves to BPS (Toray, Tokyo, Japan) were determined in rings from PH and control animals pre-contracted with 5 HT at 10^-5^ M in the absence or presence of the Kv channel antagonist 4-AP (10 mmol/L) (Sigma, United States, Cat. no. 072K3640), or the EP4 selective antagonist GW627368X (Cayman Chemical Company; Cat. no. 10009162) (10^-7^M). 5HT was selected for precontraction based on previous studies (Guibert et al., 2005; Lee et al., 2010; Liu et al., 2013) and at a concentration adapted from Takeo (1992) modified after preliminary experiments. Dose response curves were also determined for contractile responses to 4-AP.

### RNA Extraction and Purification

RNAs from Human and rats PAs were isolated by using Trizol-Reagent (Applygen, China). The tissue samples were homogenized in 10 ml Trizol reagent. Phase separation of RNA was performed by adding one-tenth volume of chloroform, vortex mixing for 15 s, and centrifuged at 12,000 × *g* for 10 min. Isopropyl alcohol (0.5 ml/1 ml Trizol) was added to the aqueous phase to precipitate total RNA. Precipitate was washed twice with 75% ethanol. For Affymetrix analysis, the RNA sample was dried and then re-dissolved. RNA quality was determined by the ratio of absorbance at 260–280 nm (A260/A280). All extracted RNA was further purified using RNeasy Clean Up kit (Qiagen) to increase A260/A280 readings.

### Quantitative Real-Time PCR

Total isolated RNA was reversely transcribed into single-stranded cDNA according to manufacturer instructions (Zhongshang Golden Bridge Company, Beijing, China). cDNA samples were used as templates for quantitative real-time PCR. Primers with specific product size were designed by Omega software based on the rat nucleotide sequences of Kv genes as the previously reported. β-actin also was used as an internal positive control (Supplementary Table S1). Quantitative real-time PCR products were separated by 1% agarose gel to verify the products size.

### PA Protein Preparation and Western Blot Analysis

Both human and rats PA tissues were thawed and homogenized in ice-cold buffer (20 mM Tris, 140 mMNaCl, 3 mM EDTA, 10 mM NaF, 10 mM sodium pyrophosphate, 2 mM NaVO4, 10% glycerol, pH7.4 and 1% TritonX-100) supplemented with protease inhibitors (content: 1.5 μM aprotinin, 20 M leupeptin, 50 M phenylmethylsulfonylfluoride, and 1.5 M benzamidine). The insoluble material was removed by centrifugation at 20,000 × *g* for 30 min at 4°C. Supernatant containing equal amounts of protein was separated by SDS-PAGE and was transferred to nitrocellulose membranes. After incubation with human polyclonal antibodies against Kv1.2, Kv1.5, and Kv2.1 receptors (1:300 dilution; Alomone Labs, Jerusalem, Israel), the blots were washed and incubated with peroxidase-conjugated secondary antibody, and protein bands were analyzed using a chemiluminescence kit (Santa Cruz Biotechnology). GAPDH was used as the loading control.

### Cell Dissociation and Hypoxic Exposure

Pulmonary artery smooth muscle cells were dissociated from fresh PA segments (further than third bifurcations in the pulmonary arterial tree) as previously described (Morrell et al., 1999). Briefly, PASMCs were isolated from precapillary pulmonary arterial vessels of adult male SD rats (200–250 g body weight). SMCs were identified by immunohistochemical staining with α-SMC (data not shown). Rat PASMC were exposed to hypoxia (1% O_2_, 5% CO_2_, rest N_2_) in 1% (v/v) FCS and 1% (m/v) penicillin and streptomycin M199 medium for the indicated period of time. All other measurements were performed under normoxic conditions, starting 30 min after termination of hypoxia.

### Whole-Cell Patch-Clamp

Kv channel currents were recorded according to the Whole-Cell Patch-Clamp technique (Ye et al., 2005). Following initial PA equilibration in hypoxic iced-low Ca^2+^ Krebs solution small pieces of muscle layers were stood for 20 min at room temperature prior to digestion with 4 mg/ml papain, 1.25 mg/ml BSA and 2 mg/ml DTT in 2 ml hypoxic low-Ca^2+^ dissolution solution. Following threefold washing in low-Ca^2+^ solution separated PASMC were stored in hypoxic low-Ca^2+^ solution with 0.5% BSA at 4°C for use within 3–4 h. Voltage clamp experiments were performed under hypoxic or normoxic conditions, as indicated. PASMC isolation for normoxic experiments were identically processed except under normoxia. Drug interventions were as indicated in Section “Results.”

All recordings were performed using an Axopatch 200B patch clamp amplifier and 3- to 5-MΩmicropipettes. PASMCs were voltage-clamped at -70 mV, and currents were evoked by steps of 200-ms duration from -70 to +70 mV. Membrane currents were filtered at 5 kHz, digitized using a Digidata 1320A interface (Axon Instruments, Foster City, CA, United States), and analyzed using pCLAMP software.

### Statistical Analysis

Values are given as mean ± SEM (standard error of the mean) or SD (standard deviation) or n. Statistical tests were two sided and data was tested for normality. For the comparison of two groups, a *t*-test was used. For more than two groups one-way ANOVA followed by Dunnett’s *post hoc* test was applied. A *P*-value of less than 0.05 was considered significant.

## Results

### Effects of Beraprost in Experimental PH

**FIGURE 1:**
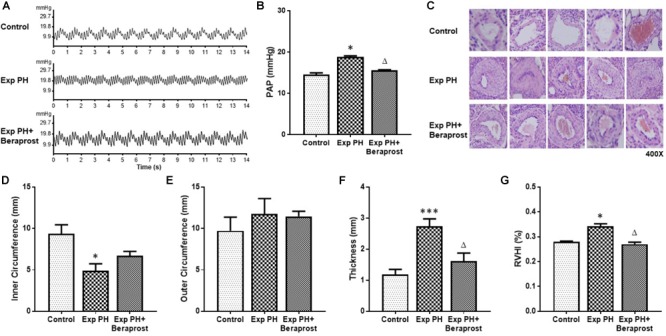
The figure shows representative traces **(A)** and mean pulmonary pressures **(B)**, representative histology (**C**, HE staining at 400x magnification) of pulmonary arteries and morphometric measures **(D–G)** in control rats (control, *n* = 12) and experimental PH rats before (*n* = 24) and after(*n* = 12) 21 days oral Beraprost treatment. RVHI was calculated as [RV weight/LV + S weight]. Results are mean ± SEM. ^∗^*p* < 0.05; ^∗∗∗^*p* < 0.001 Exp PH vs. control. ∆*p* < 0.05 Exp PH vs. Exp PH+Beraprost.

Animals and groups were as described under Methods. Experimental PH was confirmed in the hypoxic rats by HE staining and trans-catheter pulmonary pressure measurement. Abnormalities in structural proliferation [inner and outer circumference of PA, thickness of SMC layer in PA, RVHI] and PAP were all significantly rescued by BPS treatment (Figure 1).

### PGI2 and PGE2 Level in Plasma in Experimental PH and Control Rats Before and After Beraprost Treatment

**FIGURE 2:**
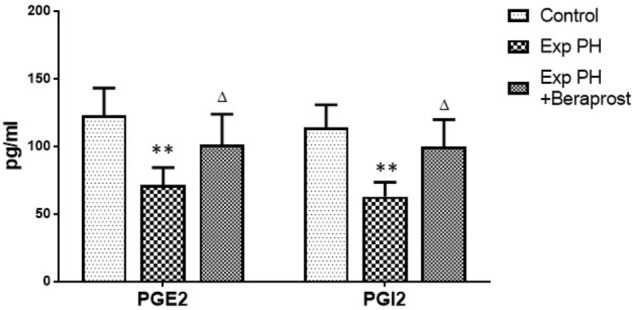
PGI2 and PGE2 levels in Plasma in control (*n* = 12) and experimental PH rats untreated (*n* = 24) and after 21 days Beraprost treatment (*n* = 12). PGI2 and PGE2 were measured by ELISA. Results are mean ± SEM. ^∗∗^*p* < 0.01 Exp PH vs. control group. ∆*p* < 0.05 Exp PH vs. Exp PH + Beraprost group.

Levels of both PGI2 and PGE2 in plasma were dramatically depressed in experimental PH rats compared with controls (*P* < 0.01). However, these depressed levels were elevated by BPS treatment (*P* < 0.05) (Figure 2).

### Gene Expression of Prostanoid Receptors in Normal Pulmonary Artery

**FIGURE 3:**
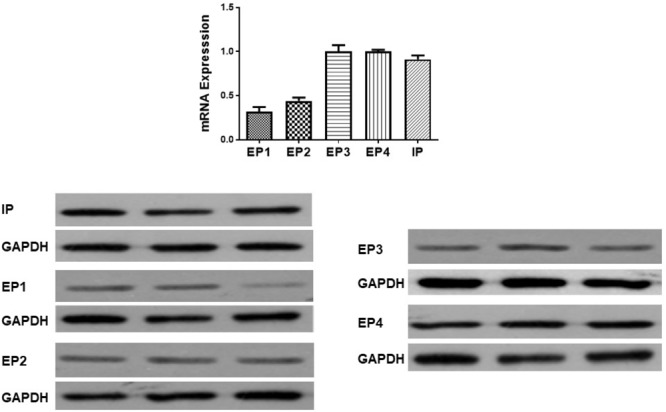
Gene and protein expression of prostanoid receptors in non-pulmonary hypertension rats. Pulmonary artery tissues were obtained from the third and subsequent bifurcations. The **(upper panel)** shows relative mRNA expression using primers shown in Supplementary Table S1. The **(lower panel)** shows Western blots of protein expression. A different gel was used for each receptor and the respective GAPDH band is shown for each. Gels were analyzed with a Biorad Universal Hood II Molecular Imager Gel System.

Gene expression of prostanoid receptors in non-PH rats was measured both by real time PCR and Western Blot. The expression of IP, EP3 and EP4 are much higher than that of EP1 and EP2 receptors in pulmonary arteries from normal pulmonary arteries (Figure 3). Thus of prostanoid receptors with a known vasodilator function, IP and EP4 are the most highly expressed.

### Expressions of EP4 and IP in Experimental PH and Control Rats and in PAH in Humans

**FIGURE 4:**
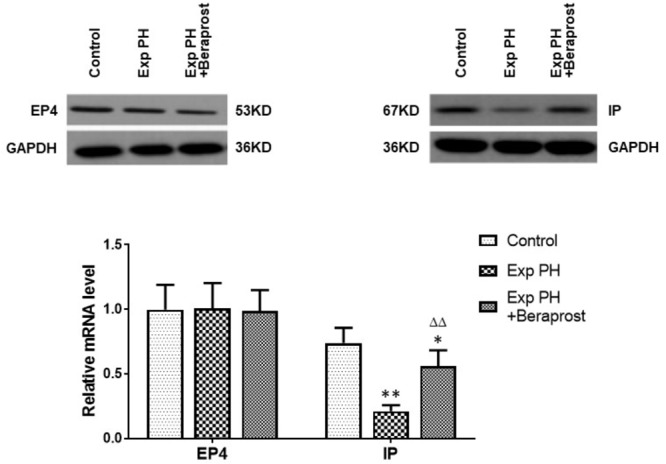
The figure shows the expression of EP4 and IP receptors in control (*n* = 12) and experimental PH rats without (*n* = 24) and following (*n* = 12) Beraprost treatment. mRNA levels were obtained by real time PCR as in Figure 3. Results are mean ± SEM. ^∗^*p* < 0.05 Exp PH + Beraprost vs. Control. ^∗∗^*p* < 0.01 Exp PH vs. Control. ^ΔΔ^*p* < 0.01 Exp PH vs. Exp PH + Beraprost.

**FIGURE 5:**
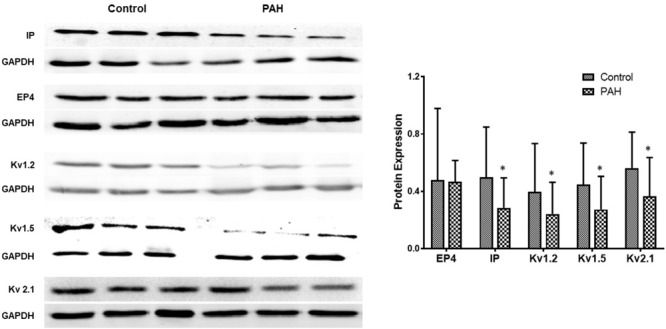
The figure shows representative Western blots of IP, EP4 receptors and Kv channels in human pulmonary arteries and mean data from non PAH (control; *n* = 11, male = 5) and PAH patients (*n* = 12, male = 6) undergoing surgery for CHD repair or lung cancer. The panel shows Western blots of protein expression. A different gel was used for each receptor and the respective GAPDH band is shown for each. Results are mean ± SD. ^∗^*p* < 0.05 PAH vs. control.

In rats the mRNA and protein expressions of the IP receptor are significantly reduced in the PH group compared with the control group, whilst EP4 expression is unchanged (Figure 4). Similar changes were found between PAH and non-PAH groups in human PAs (Figure 5 and Table 2). However, the reduced gene expression of IP receptor recovers after BPS treated in experimental PH rats (Figure 4) without change in expression of EP4 which remains at similar levels to the control animals (Figure 4).

**Table 2:** mRNA expression levels from human pulmonary arteries.

Receptor	mRNA Non-PAH	mRNA PAH
EP4	1.29 ± 1.00	1.26 ± 0.62
IP	1.58 ± 1.42	1.17 ± 0.74^∗^
Channel		
Kv1.2	1.07 ± 0.40	0.06 ± 0.03^∗∗^
Kv1.5	2.30 ± 0.94	1.12 ± 0.66^∗∗^
Kv2.1	1.15 ± 0.59	0.16 ± 0.08^∗∗^

*mRNA expression of IP, EP receptors and Kv channels in human pulmonary arteries from patients with (*n* = 11, male = 5) and without (*n* = 12, male = 6) PAH. Results were obtained from real time PCR using same primers as for rats. Results are mean ± SD. ^∗^*p* < 0.05, ^∗∗^*p* < 0.0.01 PAH vs. Non-PAH.*

### Expressions of Kv Channels in Experimental PH and Control Rats Before and After Beraprost Treatment

**FIGURE 6:**
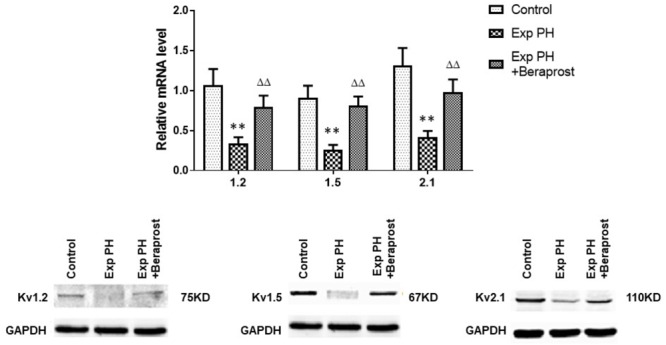
mRNA expression of Kv channels in pulmonary arteries from control rats and from experimental PH rats untreated (*n* = 24) or following Beraprost treatment (*n* = 12). mRNA expression levels were determined using real time PCR following mRNA extraction and using primers designed by Omega software based published rat nucleoside sequences of Kv genes. Results are mean ± SEM. ^∗∗^*p* < 0.01 Exp PH vs. Control. ^ΔΔ^*p* < 0.01 Exp PH vs. Exp PH + Beraprost.

Gene expressions of Kv channels, including Kv 1.5, Kv1.2, Kv2.1, are significantly lower in experimental PH than control rats and in human PAs from patients with PAH (Figures 5, 6 and Table 2). Furthermore, the decreased expression recovers with BPS treatment in experimental PH rats (Figure 6).

### Response of Pulmonary Artery Ring to EP4 Selective Antagonist GW 627368X and Beraprost in Experimental PH and Control Rats

**FIGURE 7:**
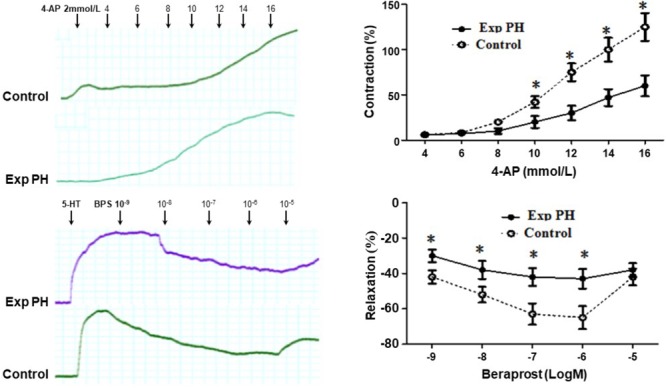
Contraction responses in pulmonary artery rings to the Kv channel antagonist 4-AP and relaxation responses to Beraprost in pulmonary artery rings from rats with (*n* = 10) and without (*n* = 10) experimental PH. Isolated pulmonary artery rings from control rats were suspended in oxygenated Krebs solution whilst those from experimental PH rats were suspended in hypoxic Krebs solution. Dilatory responses we obtained in rings pre-contracted with 5-HT (10^-5^M). Results are mean ± SEM. ^∗^*p* < 0.05 Exp PH vs. control.

**FIGURE 8:**
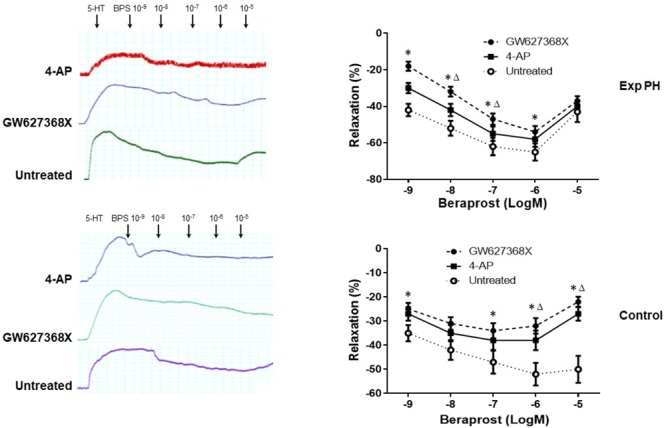
Dilatory responses in pulmonary artery rings to Beraprost in the presence or absence of the Kv channel antagonist 4-AP or the EP4 antagonist GW 627368X in rats with (*n* = 10) and without Experimental PH (*n* = 10). Results are mean ± SEM. ^∗^*p* < 0.05 Presence vs. absence of GW27368X. ^Δ^*p* < 0.05 GW27368X vs. 4-AP.

Both the contraction in response to 4-AP and the dilatation in response to BPS were much less in pulmonary artery rings from experimental PH than in those control rats (all *p* < 0.05) (Figure 7). Furthermore, the dilating responses to BPS were significantly reduced when pulmonary artery rings were pre-contracted by the EP4 selective antagonist GW 627368X or Kv channel antagonist 4-AP in both PH and control groups (all *p* < 0.05) (Figure 8).

### Kv Channel Current of PASMC in Rats Tested by Whole Cell Patch Clamp

**FIGURE 9:**
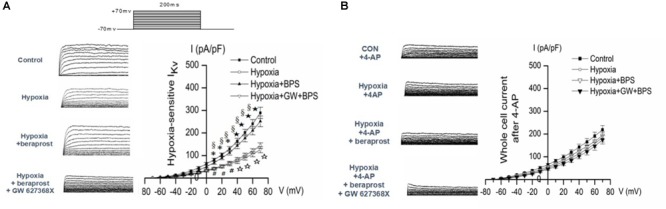
Whole-cell K^+^ current (IK) and EM of PASMCs were measured by whole cell patch clamp in normoxia (control) and hypoxia setting with or without treatment as shown. **(A)** In the absence of the Kv channel blocker 4-AP. ^∗^*p* < 0.05, ⋆*p* < 0.01 Hypoxia vs. Control. §*p* < 0.05 Hypoxia + BPS vs. Hypoxia. #*p* < 0.05, ☆*p* < 0.01 Hypoxia + GW + BPS vs. Hypoxia + BPS. **(B)** In the presence of the Kv channel blocker 4-AP. ^∗^*p* < 0.05 Hypoxia vs. Control.

Patch clamp experiments demonstrated reduced slope of the Kv current–voltage curve in PMSCs from experimental PH rats (examined under hypoxic conditions) which could be reversed in the presence of BPS. However, this effect of BPS was no longer present in the presence of the EP4 selective antagonist, GW 627368X (Figure 9A).

In order to confirm that the effects of hypoxia, BPS and GW627368X were mediated by Kv currents, patch clamp experiments were conducted in the presence or absence of the Kv channel blocker 4-AP. Effects of hypoxia, BPS and GW 627368X were all abolished in the presence of Kv channel blockade (Figure 9B).

## Discussion

In the current series of studies, we have used both an animal experimental model with PH as well as human pulmonary arteries from patients with known PAH to investigate the mechanisms of action of the oral PGI2 analogue BPS. Animals with experimental PH had lower circulating levels of PGI2 and PGE2 whilst animals with PH and humans with PAH both showed reduced vascular expression of the IP receptor. In animals this reduced expression is partially reversed by BPS treatment which also rescues the physiological phenotype. Expression of O_2_ sensitive Kv channels was reduced in animals and humans with PH and was restored by BPS treatment in animals. However, both the dilatation response of vascular rings and the magnitude of the Kv channel response to the PGI2 analog, BPS were shown to be attenuated with blockade of the EP4 receptor suggesting involvement of the EP4 receptor in mediating the effects of PGI2 on O_2_ sensitive Kv channels and vasomotion, although a contribution from off target effects could not be excluded.

The prostanoids play important roles in the regulation of vascular tone by binding to prostanoid receptors, members of the GPCRs (Smyth et al., 2009; Strassheim et al., 2018). In addition the prostanoids also act through effects on other processes such as inflammation (Clapp and Gurung, 2015). PGE2 is the most widely synthesized prostaglandin whilst PGI2 has been identified as a major vascular endothelium derived active substance responsible for dilatation in pulmonary arteries following binding with the IP receptor. In keeping with previous studies we demonstrate high expressions of IP, EP3 and EP4 receptors in pulmonary arteries from normal pulmonary arteries (Lai et al., 2008; Clapp and Gurung, 2015). EP2 receptors are predominantly located in pulmonary veins (Sugimoto and Narumiya, 2007).

Following prostanoid binding the IP receptor predominantly couples to the Gs type protein leading to an increase in cAMP, with subsequent vasodilatory and platelet anti-aggregatory effects. The effects of PGE2 are transduced by one or more of the four EP receptors (EP1, EP2, EP3, and EP4) with heterogeneity in the coupling of these receptors to intracellular signal transduction pathways (Sugimoto and Narumiya, 2007). Of the four EP receptors, the EP3 and EP4 receptors bind PGE2 with highest affinity (Kd < 1 nM), whereas the EP1 and EP2 receptors bind with lower affinity (Kd > 10 nM). EP1 activates the G protein Gq, mediating enhanced intracellular Ca^2+^ levels by influencing phosphatidylinositol turnover (Breyer et al., 2001). EP3 signaling is complex and dependent on splice variants. cAMP generation is inhibited via a Gi coupled mechanism, additional signaling mechanisms include Gs and Ca^++^ release (Breyer et al., 2001). EP2 and EP4, like IP, activate Gs and increase intracellular cAMP levels, resulting in the opening of K^+^ channels, cellular hyperpolarization, and vasodilatation (Clapp and Gurung, 2015). As confirmed in the current study EP3 and EP4 receptors are expressed in much higher concentrations than EP1 and EP2 receptors in pulmonary arteries (Sugimoto and Narumiya, 2007). Furthermore, both EP4 and IP receptors are expressed mainly in smooth muscle cells in pulmonary arteries, while EP2 receptor that mediate vasorelaxation are predominantly expressed in pulmonary veins (Walch et al., 1999). Hence, EP4 and IP are the most important receptors for pulmonary arteries dilating response of PGI2 and PGE2 with the IP receptor having a higher affinity for PGI2 than EP4. The extent to which EP4 might contribute to the vasodilatory effect of PGI2 analogues in hypoxic PH is not clear, though previous studies have indicated, a role under conditions of low IP expression in monocrotaline induced PH (Lai et al., 2008).

Reduced pulmonary production of PGI2 has been shown to occur in PH and be due, at least in part, to reduced activity of prostaglandin synthase (Tuder et al., 1999). In the current study there was a substantial reduction in the circulating level of PGI2 (and PGE2) in the chronically hypoxic rat model prior to treatment with subsequent restoration. The mechanism for this restoration is not apparent but likely involves changes in vascular cells with chronic treatment given the distribution of prostaglandin synthase (Gryglewski, 2008).

Previous studies have also demonstrated a reduction in IP receptor expression in both animal models and in humans with PAH which was confirmed in the current study (Mason et al., 1999; Hoshikawa et al., 2001; Lai et al., 2008). Interestingly in the animal model then treated with BPS there was a substantial recovery in IP receptor expression. This differs from previous findings in children with PAH in whom IP receptor expression in intra acinar arteries, but not in pre acinar, was reduced in those who had been treated with prostacyclin (Falcetti et al., 2010) as has been observed elsewhere in relation to the IP receptor (Adie et al., 1992). The usual pharmacological response to increased exposure to agonist is a receptor down regulation. Although examples of agonist induced receptor upregulation exists (Thomas et al., 1992). However a number of studies with other GPRCs have shown that the initial down-regulation is then followed by a recovery in receptor expression which appears to require protein synthesis (Bengtsson et al., 1996). The mechanism for the effect observed in the present study is not known, in particular whether the recovery in receptor expression could be related to the restoration in plasma levels of PGI2 and PGE2, but a recovery in IP receptor expression would contribute to the beneficial effects of BPS therapy.

Consistent with the down-regulation of the IP receptor in PH we observed that the dilating effect of BPS was much less on pulmonary artery rings from PH than on ones from Non-PH control rats. However, we also found that the effects of BPS were also significantly reduced when pulmonary artery rings pre-contracted by EP4 selective antagonist GW 627368X not only in PH but also control groups. Thus it seems that the beneficial effects in hypoxic PH of BPS may, at least in part, be occurring through binding to the EP4 receptor which may overcome the reduction in expression of the IP receptor as also seen in monocrotaline induced PH (Lai et al., 2008). PGI2 cross binding and signal transduction through the EP4 receptor has been previously reported in other tissues (Davis et al., 2004; Lin et al., 2006). These studies suggest the possibility that a specific E4 agonist may be of therapeutic values in human PAH.

A much less well explored pathway is the possible effect of PGI2 on oxygen sensitive Kv channels which are themselves thought to play a role in the development of PAH (Bonnet et al., 2006). It is known that such channels are preferentially expressed in resistance rather than conduit pulmonary arteries (Archer et al., 2004). Their normal physiological function is to increase pulmonary vascular resistance in the fetal circulation to divert blood through the patent ductus arteriosus whilst in adults they likely contribute to ventilation –perfusion matching. They comprise a number of heterologous receptors which are inhibited at low oxygen tensions leading to inhibition of the outward potassium current and consequent depolarization (Patel and Honore, 2001). Calcium influx in response to depolarization through voltage gated and other calcium channels then leads to increase in intracellular Ca^2++^ and vasoconstriction. In the current study we confirmed/demonstrated a reduction in mRNA and protein expression of the O_2_ sensitive Kv1.2, 1.5, and 2.1 channels in both humans with PAH and animal model with PH. As with the IP receptor, BPS treatment was able to rescue this phenotype in the experimental model.

Patch clamp experiments demonstrated reduced function of the hypoxia sensitive Kv channels in PASMCs from PH rats and, in keeping with the effects on Kv channel expression these were reversed by BPS. This effect of BPS was prevented in the presence of Kv blockade with 4 AP. However, the effect of BPS was also prevented by blockade of the EP-4 receptor. This indicates that not only is there a link between prostanoid pathways and O_2_ sensitive Kv channels but also a new mechanism of IP agonist, such as BPS, modulating O_2_ sensitive Kv channels in PH.

## Conclusion

In conclusion, this study indicates that the salutatory effects of the IP2 analog, BPS, on vascular contraction in PAH may in part be mediated by binding to the EP4 receptor and restoring the function of O_2_ sensitive Kv channels. Further studies are required to directly prove the interaction of BPS and the EP4 receptor.

## Ethics Statement

The use of the animals were in accordance to the Basel Declaration and was approved by the Animal Experimentation Committee of Medical College, Xi’an Jiaotong University, China. Written informed consent was obtained from all patients in accordance with the Declaration of the Helsinki. The study was approved by the ethics committee of the First Affiliated Hospital of Xi’an Jiaotong University, China.

## Author Contributions

FF contributed to study design, experiments control, analysis and writing of the manuscript. HT major contributor for the molecular and patch clamp experiments. JG major contributor for vessel ring and partial in the molecular experiments. JD major contributor in human tissue collection, experiments and parts of the molecular experiments. YL involved in molecular and vessel ring experiments and data analysis. CC involved in molecular and patch clamp experiments and data analysis. SZ involved in tissue and blood collection. YsZ involved in study design, experiments control, and analysis. JL involved in human tissue collection, consents and the molecular experiments. HyT involved in study design, experiments control, and analysis. AD contributed in interpretation and major contributor in writing of the manuscript and making final decision to submit for publication. YlZ contributed in study design, patient consent and human sample collection. All authors read and approved the final manuscript.

## Conflict of Interest Statement

The authors declare that the research was conducted in the absence of any commercial or financial relationships that could be construed as a potential conflict of interest.

## Supplementary Material

TABLE S1 Primer sequences used for real time PCR.

## Abbreviations

4-AP: 4-aminopyridine
AC: adenyl cyclase
BPS: Beraprost
PGE2: E type prostaglandin
EP: E type prostaglandin receptor
GPCR: G protein coupled receptors
IK: S: interventricular septum: K^+^ current
IP: PGI2 receptor
Kv: voltage-gated K^+^ channels
LV: left ventricle
PAH: pulmonary arterial hypertension
PAP: pulmonary artery pressure
PASMC: pulmonary artery smooth muscle cell
PGI2: prostacyclin2
PH: pulmonary hypertension
RVHI: right ventricle hypertrophy index
