# Supplementary material for: Mechanism of Beraprost Effects on Pulmonary Hypertension: Contribution of Cross-Binding to PGE2 Receptor 4 and Modulation of O2 Sensitive Voltage-Gated K+ Channels
Source: Front Pharmacol. 2019 Jan 18;9:1518. doi: 10.3389/fphar.2018.01518 (PMC6346678; doi:10.3389/fphar.2018.01518)
Supplement: TABLE S1 — Primer sequences used for real time PCR. [file Presentation_1.PPTX]

## Slide 1
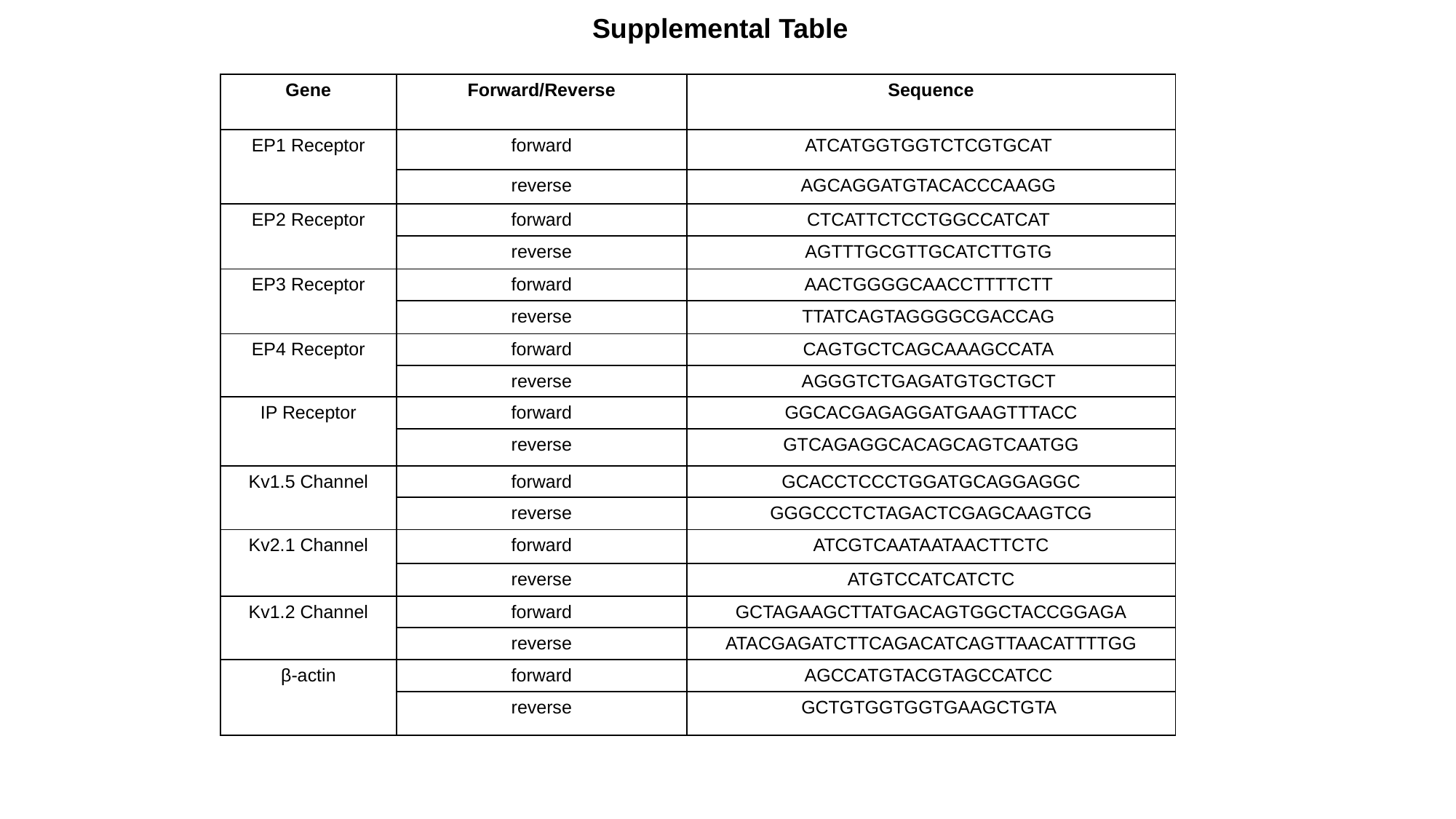

Supplemental Table
| Gene | Forward/Reverse | Sequence |
| --- | --- | --- |
| EP1 Receptor | forward | ATCATGGTGGTCTCGTGCAT |
| | reverse | AGCAGGATGTACACCCAAGG |
| EP2 Receptor | forward | CTCATTCTCCTGGCCATCAT |
| | reverse | AGTTTGCGTTGCATCTTGTG |
| EP3 Receptor | forward | AACTGGGGCAACCTTTTCTT |
| | reverse | TTATCAGTAGGGGCGACCAG |
| EP4 Receptor | forward | CAGTGCTCAGCAAAGCCATA |
| | reverse | AGGGTCTGAGATGTGCTGCT |
| IP Receptor | forward | GGCACGAGAGGATGAAGTTTACC |
| | reverse | GTCAGAGGCACAGCAGTCAATGG |
| Kv1.5 Channel | forward | GCACCTCCCTGGATGCAGGAGGC |
| | reverse | GGGCCCTCTAGACTCGAGCAAGTCG |
| Kv2.1 Channel | forward | ATCGTCAATAATAACTTCTC |
| | reverse | ATGTCCATCATCTC |
| Kv1.2 Channel | forward | GCTAGAAGCTTATGACAGTGGCTACCGGAGA |
| | reverse | ATACGAGATCTTCAGACATCAGTTAACATTTTGG |
| β-actin | forward | AGCCATGTACGTAGCCATCC |
| | reverse | GCTGTGGTGGTGAAGCTGTA |
